# Improvement of COVID‐19 with renal failure and paralytic ileus by using steroids

**DOI:** 10.1002/rcr2.689

**Published:** 2020-11-29

**Authors:** Atsushi Kitamura, Clara So, Torahiko Jinta

**Affiliations:** ^1^ Department of Pulmonary Medicine Thoracic Center, St. Luke's International Hospital Tokyo Japan

**Keywords:** Acute kidney injury, COVID‐19, paralytic ileus

## Abstract

A 51‐year‐old man attended our hospital with chief complaints of fever and diarrhoea for the past eight days. Chest computed tomography showed peripherally dominant ground‐glass opacity. Severe acute respiratory syndrome coronavirus 2 (SARS‐CoV‐2) RNA was detected by real‐time polymerase chain reaction, and the patient was diagnosed with coronavirus disease (COVID‐19). His clinical course included respiratory failure, acute kidney injury, and paralytic ileus. Systemic management was difficult, but he recovered with high‐dose steroids, temporary haemodialysis therapy, and a nasointestinal tube, without antiviral drugs. COVID‐19 can be associated with multiple organ failure due to vascular endothelial injury.

## Graphical abstract

Coronavirus disease (COVID‐19) may be associated with multiple organ failure due to vascular endothelial damage. Here, we report a case of COVID‐19 in which a patient suffered from ileus complications and was difficult to manage systemically. However, he recovered with high doses of steroids, hemodialysis, and the nasal intestine without the use of antivirals.

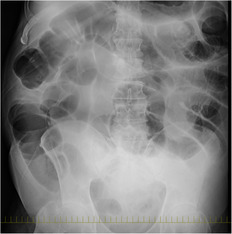

## Introduction

Coronavirus disease (COVID‐19) can be associated with multiple organ failure due to vascular endothelial injury [1]. In such cases, there can be respiratory failure, paralytic ileus, and acute renal failure. Here, we report a case of COVID‐19 in which the patient had ileus complications and was difficult to manage systemically; however, he recovered with high‐dose steroid administration, haemodialysis therapy, and a nasointestinal tube, without the use of antiviral drugs.

## Case Report

**Figure 1:**
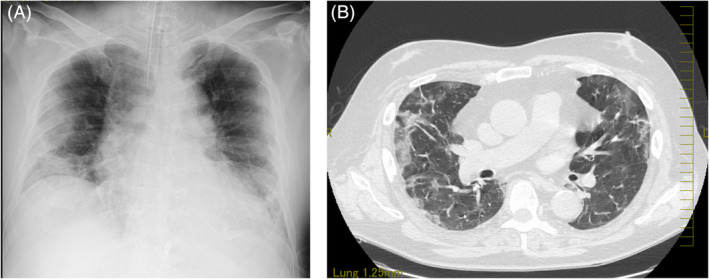
(A, B) X‐ray and computed tomography (CT) of the chest on admission demonstrated diffuse ground‐glass opacity located predominantly at the pulmonary periphery.

A 51‐year‐old man was admitted to our hospital with a history of persistent fever and diarrhoea for the past eight days. He had a smoking history of seven pack‐years and consumed alcohol occasionally; his past and family histories were non‐contributory. Physical examination on admission revealed a fever of 39.0°C, moist skin, and coarse crackles in the lower chest bilaterally. Complete blood counts and serum chemistry suggested a serious infectious event, with a markedly elevated serum leucocyte count of 13,000/μL with a left‐shifted haemogram, C‐reactive protein of 12.7 mg/dL, elevation of lactate dehydrogenase to 430 U/L (normal: <223 U/L), elevation of aspartate aminotransferase to 41 U/L, elevation of alanine aminotransferase to 49 U/L, serum creatinine of 3.19 mg/ dL, serum uric acid of 110.4 mg/dL, and plasma D‐dimer of 2.5 μg/mL. A haemoglobin A1c value of 6.5% and mild glucose intolerance were found. Urinalysis showed protein 3+, uric blood −, and urine sediment of 5+ granular casts, 4+ epithelial casts, and 5+ hyaline casts. A nasopharyngeal swab was positive for severe acute respiratory syndrome coronavirus (SARS‐CoV‐2) RNA on real‐time polymerase chain reaction (PCR). Chest X‐ray and chest computed tomography (CT) on admission demonstrated diffuse ground‐glass opacity located predominantly at the periphery of the lower lung lobes (Fig. 1). Abdominal contrast‐enhanced CT showed no obvious abdominal abnormalities, including in the intestinal tract.

On the basis of the above typical imaging findings and the positive PCR, respiratory failure and renal failure due to COVID‐19 were diagnosed.

**Figure 2:**
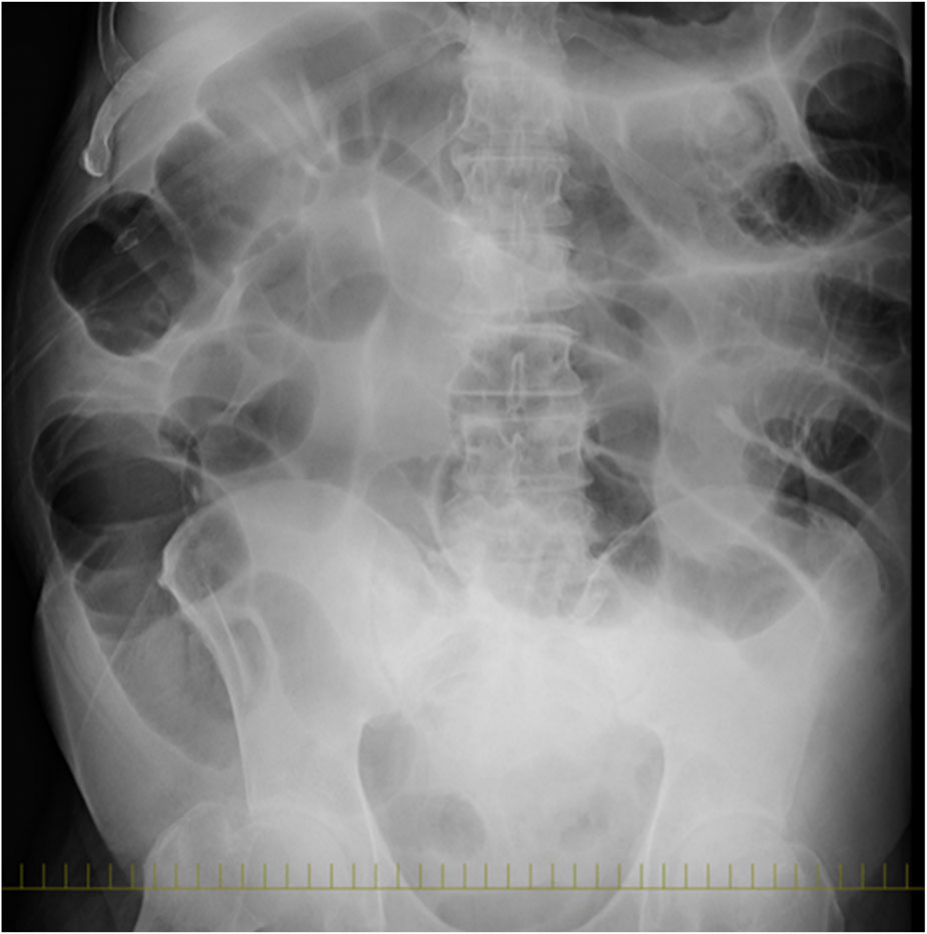
X‐ray of the abdomen demonstrated dilatation of the intestinal tract.

After admission, the patient was unable to maintain 90% SpO_2_ (peripheral capillary oxygen saturation), even with 10 L/min oxygen supplied by a non‐rebreather mask, and he was intubated on a ventilator. Methylprednisolone 500 mg was intravenously administered once daily for three days. The steroid was then changed to oral prednisolone, which was given every second day at reducing doses of 60, 40, 20, 10, and 5 mg. Because bacterial pneumonia with viral pneumonia could not be ruled out, tazobactam− piperacillin 13.5 g/day and levofloxacin 500 mg/day were administered. Unfractionated heparin at 10,000 units/day was administered by subcutaneous injection to prevent thrombus formation. The patient was extubated on day 4 after intubation as his respiratory condition improved. On day 5, his serum creatinine had risen to 11.13 mg/dL and his urine volume was less than 500 mL/day, so temporary haemodialysis was performed on days 5, 6, 8, and 9. After extubation, tube feeding was continued, but vomit‐like bile was found on day 8, and an abdominal X‐ray photograph showed dilatation of the intestinal tract (Fig. 2). Abdominal contrast‐enhanced CT showed no intestinal obstruction, and functional (paralytic) ileus was diagnosed. SARS‐CoV‐2‐PCR on the stool was not collected, but he had diarrhoea from the admission and was diagnosed with ileus due to COVID‐19. The patient was treated conservatively, with discontinuation of solid food intake, total parenteral nutrition (TPN) management, and insertion of a nasointestinal tube to keep the intestinal tract at rest. The substantial fluid loss from the intestinal tract made it difficult to manage the TPN. From day 20, drainage from the nasointestinal tubes began to decrease, kidney function improved, urine output increased, and the patient began a liquid diet. Liver function peaked on the first day and improved thereafter. The diet was gradually stepped up to solids, and the man was discharged on day 38 after confirmation that there was no relapse of abdominal symptoms.

## Discussion

In this patient, COVID‐19 infection was accompanied by multiple organ failure, including respiratory failure, paralytic ileus, and acute renal failure. SARS‐CoV‐2 infection in humans is known to be mediated through binding of the virus to the angiotensin‐converting enzyme‐2 (ACE2) receptor on cell surfaces. It was recently found that the ACE2 receptor is also expressed on vascular endothelial cells, and SARS‐CoV‐2 can infect these cells via the receptor. Vascular endothelial cell injury can lead to multiple organ damage due to microthrombus formation in the cardiovascular system [1]. In light of the patient's lack of intestinal obstruction on CT and the course of his clinical recovery, we consider that the renal injury—and especially the paralytic ileus—in this case may have been functional injuries due to vascular‐mediated organ ischaemia. In addition, PCR for stools was possible at the laboratory in Japan at that time, but it was impossible to test in clinical practice, so it was not collected. Colonoscopy was not performed due to the exposure of the examining physician to the virus. Acute Respiratory Distress Syndrome (ARDS) with COVID‐19 is reported to have more complications of ileus than ARDS without COVID‐19 [2]. As he had diarrhoea from the beginning of his admission and later recovered with improvement of his general condition, we thought that the symptoms were caused by the viruses.

Renal injury occurs in 75.4% of COVID‐19 patients, with a median duration of 12 days; 45.7% of patients with renal injury from COVID‐19 recover completely [3]. Owing to complications of his ileus, the patient had substantial fluid loss from the intestinal tract; this made it difficult to manage his TPN and delayed the recovery of renal function. Gastrointestinal symptoms, including nausea and vomiting, are relatively common, occurring in 50.5% of COVID‐19 patients [4], ileus is not rare [2].

Rapid disease exacerbation because of cytokine storm seven to 10 days after symptom onset has been suggested to occur in COVID‐19 patients [5]. From the beginning of the epidemic, we have been administering steroids to suppress the cytokine storm in cases of severe respiratory failure requiring intubation, and we have been giving subcutaneous heparin injections for anticoagulation. Although the successful use of tocilizumab for cytokine storms has been reported, the drug needs to be used carefully in diverticulitis, and it therefore would have required careful use in our patient because of his bowel dilation. The patient was successfully treated with glucocorticosteroids with no significant side effects.

Although some success with antiviral drugs such as favipiravir has been reported, this patient had renal failure and the dosage was unknown in renal failure, so favipiravir was not used. Nevertheless, we were able to cure the patient with glucocorticosteroids.

This case was complicated by a variety of organ damage caused by COVID‐19 and was difficult to manage systemically, but they recovered with supportive care and the use of systemic corticosteroids.

### Disclosure Statement

Appropriate written informed consent was obtained for publication of this case report and accompanying images.
